# Urinary and Sexual Impact of Robotic Radical Prostatectomy: Reporting of Patient-reported Outcome Measures in the First Year after Radical Prostatectomy in a Contemporary Multicentre Cohort in the United Kingdom

**DOI:** 10.1016/j.euros.2024.05.003

**Published:** 2024-05-21

**Authors:** Joshua Bridge, Muhieddine Labban, Alexander P. Cole, Busola Adebusoye, Sarah C. Smith, Evangelia Protopapa, Neil McCartan, Chris Brew-Graves, Quoc-Dien Trinh, Kevin Hamer, Sue Mallett, Jan van der Meulen, Caroline M. Moore, TrueNTH Post Surgery UK Investigators

**Affiliations:** a Division of Surgical & Interventional Science, University College London, London, UK; b National Cancer Imaging Translational Accelerator, Division of Medicine, University College of London, London, UK; c Division of Urological Surgery and Center for Surgery and Public Health, Brigham and Women’s Hospital, Harvard Medical School, Boston, MA, USA; d Department of Health Services Research & Policy, London School of Hygiene & Tropical Medicine, London, UK; e Our Future Health, London, UK; f My Medical Records, University Hospital Southampton, Southampton, UK; g Department of Urology, University College London Hospitals Trust, London, UK

**Keywords:** Prostate cancer, Patient-reported outcome measures, Radical prostatectomy

## Abstract

**Background and objective:**

Radical prostatectomy (RP) is an established treatment for localised prostate cancer that can have a significant impact on urinary and sexual function, with recovery over time. Our aim was to describe functional recovery in the first year after RP, reporting descriptive outcomes alongside validated patient-reported outcome measure scores (Expanded Prostate Cancer Index Composite, EPIC-26).

**Methods:**

Men undergoing RP between September 2015 and November 2019 completed EPIC-26 at baseline and 1, 3, 6, and 12 mo.

**Key findings and limitations:**

Overall, 2030 men consented to participation, underwent RP, and completed EPIC-26. At baseline, 97% were pad-free (1928/1996; 95% confidence interval [CI] 96–97%) and 77% were leak-free and pad-free (1529/1996; 95% CI 75–78), with a median EPIC-26 incontinence domain score of 100 (interquartile range [IQR] 86–100). At 12 mo, 65% were pad-free (904/1388; 95% CI 63–68%) and 42% were leak-free and pad-free (583/1388; 95% CI 39–45%), with a median EPIC-26 score of 76 (IQR 61–100). While one in three men reported wearing a pad at 12 mo, fewer than one in ten men needed more than 1 pad/d. At baseline, 1.9% reported a “moderate or big problem” with urine leakage, which increased to 9.7% at 12 mo. At baseline, the median sexual domain score among 1880 men was 74 (IQR 43–92) and 52% had erections sufficient for intercourse without medication (975/1880; 95% CI 50–54%). Among these 975 men, 630 responded at 12 mo, of whom 17% reported sufficient erections for intercourse (105/630; 95% CI 14–20%), without medication in 6% (37/630; 95% CI 4–8%) and needing medication in 11% (68/630; 95% CI 9–13%); the median EPIC-26 domain score was 26 (IQR 13–57).

**Conclusions and clinical implications:**

Reporting of functional outcomes after RP in terms of easily understood concepts such as pad-free and leak-free status, and erections with and with medication, alongside the classical report using EPIC-26 domain scores, increases the understanding of RP recovery patterns over the first year.

**Patient summary:**

At 12 months after surgery for prostate cancer, one in ten men reported a moderate or big problem with urine leakage and one in five men reported sufficient erections.

## Take Home Message

Approximately half of men in a contemporary UK cohort reported that they either leaked urine or used pads for urine leakage or both at 1 yr after radical prostatectomy (RP). The majority of those wearing pads needed only 1 pad per day, and only one in ten men considered urine leakage a moderate or big problem. Among men who did not need medication for erections before RP, 17% reported that they were able to achieve erections, with or without medication, at 1 yr after surgery.

## 1 Introduction

The impact of radical prostatectomy (RP) on urinary and sexual function is reported using a range of tools and thresholds at different time points. The correlation between median domain scores and descriptive terms for leakage, pad use, and ability to achieve erection are often poorly understood [1].

Here we describe functional recovery in the first year after RP using descriptive outcomes for urine leakage, pad use, problems with leakage, and ability to achieve erections, alongside median Expanded Prostate Cancer Index Composite (EPIC-26) domain scores for urinary incontinence and sexual function.

We analysed data from 26 UK centres for 1118 patients who underwent RP, of which 96% (*n* = 1069) were robot-assisted laparoscopic RP. Throughout the study we aimed to use plain English terminology to improve clarity for patients and the clinical teams advising patients. We also used multiple imputation to assess the impact of missing data on the results.

## 2 Patients and methods

### 2.1 Data source

Men scheduled for RP between November 2015 and September 2019 consented to use an online form and were prompted by the My Medical Record system to provide patient-reported outcome measure (PROM) responses at baseline and at 1, 3, 6, and 12 mo after surgery. Clinical teams contributed clinical data via the My Medical Record portal.

The PROMs included the validated EPIC-26 questionnaire [2].

### 2.2 Descriptions of urinary and sexual function

Descriptive outcomes for leak-free and pad-free status were derived from responses for the EPIC-26 incontinence domain, alongside scores for problems with urine leakage. For sexual function, the descriptive outcomes reported were “erections adequate for intercourse” with or without use of either medication or devices (Table 1). The EPIC-26 domain scores were reported on a scale from 0 to 100 for urinary incontinence and sexual function, with higher scores indicating better function [2].

**Table 1:** Descriptions of urinary and sexual functions derived from EPIC-26 domain questions

	**EPIC-26 items**	**Response**
**Urinary outcomes**		
Leak-free	“Over the past 4 weeks, how often have you leaked urine?”	“Rarely or never”
Pad-free	“How many pads or adult diapers per day did you usually use to control leakage during the last 4 weeks?”	“None”
Leak-free and pad-free	“Over the past 4 weeks, how often have you leaked urine?”	“Rarely or never”
	“How many pads or adult diapers per day did you usually use to control leakage during the last 4 weeks?”	“None”

**Sexual** o**utcomes**		
Erections adequate for intercourse without assistive medications or devices	“How would you describe the usual QUALITY of your erections during the last 4 weeks?”	“Firm enough for intercourse”
	“Have you used any medications or devices to aid or improve erections?”	“No”
Erections adequate for intercourse with assistive medications or devices	“How would you describe the usual QUALITY of your erections during the last 4 weeks?”	“Firm enough for intercourse”
	“Have you used any medications or devices to aid or improve erections?”	“Yes”
Inadequate erections	“How would you describe the usual QUALITY of your erections during the last 4 weeks?”	“Firm enough for masturbation or foreplay” **OR** “Not firm enough for any sexual activity” **OR** “None at all”

EPIC = Expanded Prostate Cancer Index Composite.

### 2.3 Demographic and clinical data

Demographic data including age, race, marital status, and comorbidities were collected directly from patients, with clinical details reported by clinical teams.

### 2.4 Statistical analysis

The analytic cohort consisted of men who underwent RP and completed a PROM at baseline and at least one time point after surgery.

We calculated median EPIC-26 scores for incontinence and sexual function domains with the interquartile range (IQR), and the proportion of men who were leak-free, pad-free, or leak-free and pad-free at each time point. The proportion of men with erections adequate for intercourse with or without medication or devices and of men with inadequate erections were recorded at each time point alongside the 95% confidence interval (CI) using the Wilson method [3].

Statistical analysis was performed using R. As a sensitivity analysis, we imputed missing data to compare with the complete-case results [4]. These results are reported in the Supplementary material.

Ethical approval was obtained from the South Central Hampshire B Research Ethics Committee (September 4, 2015; REC reference 15/SC/045, IRAS project ID 169848).

## 3 Results

A total of 2081 men gave consent to study participation. Ten men did not report demographic data, and a further ten had no PROM data. A further 31 men (5%) had only clinical data and no PROM data.

For analysis, 2030 (98%) men had baseline PROM data. Of these, 912 (45%) underwent RP at one of the ten hospitals that did not report clinical data (Supplementary Fig. 1). There were 26 hospitals and 35 named surgeons in the cohort. The surgeon and surgical approach were collected in the clinical data, which was provided by the hospitals for 1118 patients. The centre was named for 2030 patients and the surgeon was named for 847 patients. The surgical approach was reported as robotic for 1069/1118 cases, laparoscopic for 41/1118, open for 2/1118, and other for 2/1118, and was missing for 4/1118 cases. Baseline characteristics are presented in Table 2.

**Table 2:** Characteristics of participants at baseline a

	Clinical + PROM data (*N* = 1118)	PROM data only (*N* = 912)	All participants (*N* = 2030)
Mean age at surgery, yr (range)	64 (40–79)	64 (36–83)	64 (36–83)
Age group at surgery, *n* (%)			
<51 yr	39 (3)	27 (3)	66 (3)
51 yr to <61 yr	322 (29)	230 (25)	552 (27)
61 yr to <71 yr	584 (52)	492 (54)	1076 (53)
71 yr to <81 yr	156 (14)	106 (12)	262 (13)
≥81 yr	0 (0)	1 (<1)	1 (<1)
Unknown	17 (2)	56 (6)	73 (4)

Surgery date, *n* (%)			
2015	6 (1)	2 (<1)	8 (<1)
2016	227 (20)	99 (11)	326 (16)
2017	333 (30)	216 (24)	549 (27)
2018	384 (34)	352 (39)	736 (36)
2019	155 (14)	190 (21)	345 (17)
Unknown	13 (1)	53 (6)	66 (3)

Ethnicity, *n* (%)			
English/Welsh/Scottish/Northern Irish/British	948 (85)	828 (91)	1776 (87)
Any other White background	33 (3)	12 (1)	45 (2)
African	32 (3)	12 (1)	44 (2)
Caribbean	27 (2)	12 (1)	39 (2)
Irish	19 (2)	12 (1)	31 (2)
Indian	13 (1)	9 (1)	22 (1)
Any other Asian background	7 (1)	0 (0)	7 (<1)
Any other ethnic group	7 (1)	2 (<1)	9 (<1)
White and Black African	6 (1)	3 (<1)	9 (<1)
Any other Black/African/Caribbean background	5 (<1)	2 (<1)	7 (<1)
Pakistani	4 (<1)	3 (<1)	7 (<1)
White and Black Caribbean	4 (<1)	1 (<1)	5 (<1)
White and Asian	3 (<1)	2 (<1)	5 (<1)
Any other mixed/multiple ethnic background	2 (<1)	4 (<1)	6 (<1)
Arab	2 (<1)	2 (<1)	4 (<1)
Chinese	2 (<1)	3 (<1)	5 (<1)
Bangladeshi	1 (<1)	0 (0)	1 (<1)
Not available	3 (<1)	5 (1)	8 (<1)

Marital status, *n* (%)			
Married	954 (85)	795 (87)	1749 (86)
Single	157 (14)	109 (12)	266 (13)
Unknown	7 (1)	8 (1)	15 (1)

Comorbidities, *n* (%)			
Diabetes	76 (7)	60 (7)	136 (7)
Heart disease	45 (4)	54 (6)	99 (5)

Urinary leakage, *n* (%) (Over the past four weeks how often have you leaked urine?)			
Rarely or never	861 (77)	694 (76)	1555 (77)
About once a week	95 (8)	80 (9)	175 (9)
More than once a week	54 (5)	43 (5)	97 (5)
About once a day	56 (5)	48 (5)	104 (5)
More than once a day	50 (4)	41 (4)	91 (4)
Unknown	2 (<1)	6 (1)	8 (<1)

Pad use, *n* (%) (How many pads per day did you usually use to control leakage during the last 4 weeks?)			
None	1076 (96)	881 (97)	1957 (96)
1 per day	23 (2)	21 (2)	44 (2)
2 per day	7 (1)	4 (<1)	11 (1)
3 or more per day	9 (1)	5 (1)	14 (1)
Unknown	3 (<1)	1 (<1)	4 (<1)

Pad-free and leak-free, *n* (%)			
Yes	856 (77)	691 (76)	1547 (76)
No	257 (23)	215 (24)	472 (23)
Unknown	5 (<1)	6 (1)	11 (1)

Median EPIC-26 incontinence domain score (IQR)	100 (86–100)	100 (86–100)	100 (86–100)
Respondents, *n* (%)	1100 (98)	896 (98)	1996 (98)

Sexual function (How would you describe the usual quality of your erections during the last 4 weeks?)			
Firm enough for intercourse without assistance	569 (51)	433 (47)	1002 (49)
Firm enough for intercourse with medical or assistive devices	54 (5)	47 (5)	101 (5)
Not firm enough for intercourse	460 (41)	387 (42)	847 (42)
Unknown	35 (3)	45 (5)	80 (4)

Median EPIC-26 sexual domain score (IQR)	75 (45–92)	70 (39–92)	71 (42–92)
Patients, *n* (%)	1079 (97)	874 (96)	1953 (96)

Prostate-specific antigen category, n (%)			
<10 ng/ml	764 (68)	–	–
10–20 ng/ml	248 (22)	–	–
>20 ng/ml	60 (5)	–	–
Unknown	46 (4)	–	–

Gleason score, *n* (%)			
6	87 (8)	–	–
7	906 (81)	–	–
≥8	100 (9)	–	–
Unknown/missing	25 (2)	–	–

Nerve-sparing approach, *n* (%)			
None	312 (28)	–	–
Unilateral	268 (24)	–	–
Bilateral	383 (34)	–	–
Unknown	155 (14)	–	–

Lymphadenectomy, *n* (%)			
No	761 (68)	–	–
Yes	334 (30)	–	–
Unknown/missing	23 (2)	–	–

D’Amico risk category, *n* (%)			
Low	48 (4)	–	–
Intermediate	256 (23%)	–	–
High	625 (56%)	–	–
Unknown	189 (17%)	–	–

EPIC = Expanded Prostate Cancer Index Composite; IQR = interquartile range; PROM = patient-reported outcome measure.

a Percentages may not add to 100 because of rounding.

The cohort of men with PROM data at baseline and 12 mo was used to determine urinary incontinence outcomes for 1388 men and sexual function outcomes for 1200 men at 12 mo (Table 3).

**Table 3:** Urinary and sexual outcomes at 12 mo after radical prostatectomy for patients who provided baseline and 12-mo outcome data for the urinary domain (*N* = 1388) and the sexual domain (*N* = 1200)^a^

Outcome	Patient group		
**Urinary outcomes**	**Patients who provided** **urinary PROM data** **(*N* = 1388)**	**Patients who were** **leak-free and pad-free** **at baseline (*N* = 1086)**	**Patients who were not** **leak-free or pad-free** **at baseline (*N* = 302)**
Leak-free	619/1388 (45%, 42–47%)	549/1086 (51%, 48–54%)	70/302 (23%, 19–28%)
Pad-free	904/1388 (65%, 63–68%)	758/1086 (70%, 67–73%)	146/302 (48%, 43–54%)
Leak-free and pad-free	583/1388 (42%, 39–45%)	523/1086 (48%, 45–51%)	60/302 (20%, 16–25%)
EPIC-26 incontinence domain score	76 (61–100)	85 (65–100)	65 (52–79)

**Sexual outcomes**	**Patients who provided** **sexual PROM data** **(*N* = 1200)**	**Patients with natural** **erections at baseline** **(*N* = 630)**	**Patients without natural** **erections at baseline** **(*N* = 570)**
Unassisted firm erections adequate for intercourse	42/1200 (4%, 3–5%)	37/630 (6%, 4–8%)	5/570 (1%, 0–2%)
Assisted firm erections adequate for intercourse	83/1200 (7%, 6–9%)	68/630 (11%, 9–14%)	15/570 (3%, 2–4%)
Inadequate erections	1075/1200 (90%, 88–91%)	525/630 (83%, 80–86%)	550/570 (97%, 95–98%)
EPIC-26 sexual domain score	29 (18–46)	33 (21–60)	25 (18–36)

EPIC = Expanded Prostate Cancer Index Composite; PROM = patient-reported outcome measure.

a Percentages may not add to 100 because of rounding. Results are presented as *n/N* (%, 95% confidence interval), or the median score (interquartile range).

### 3.1 Analysis of urinary outcomes

**Fig. 1:**
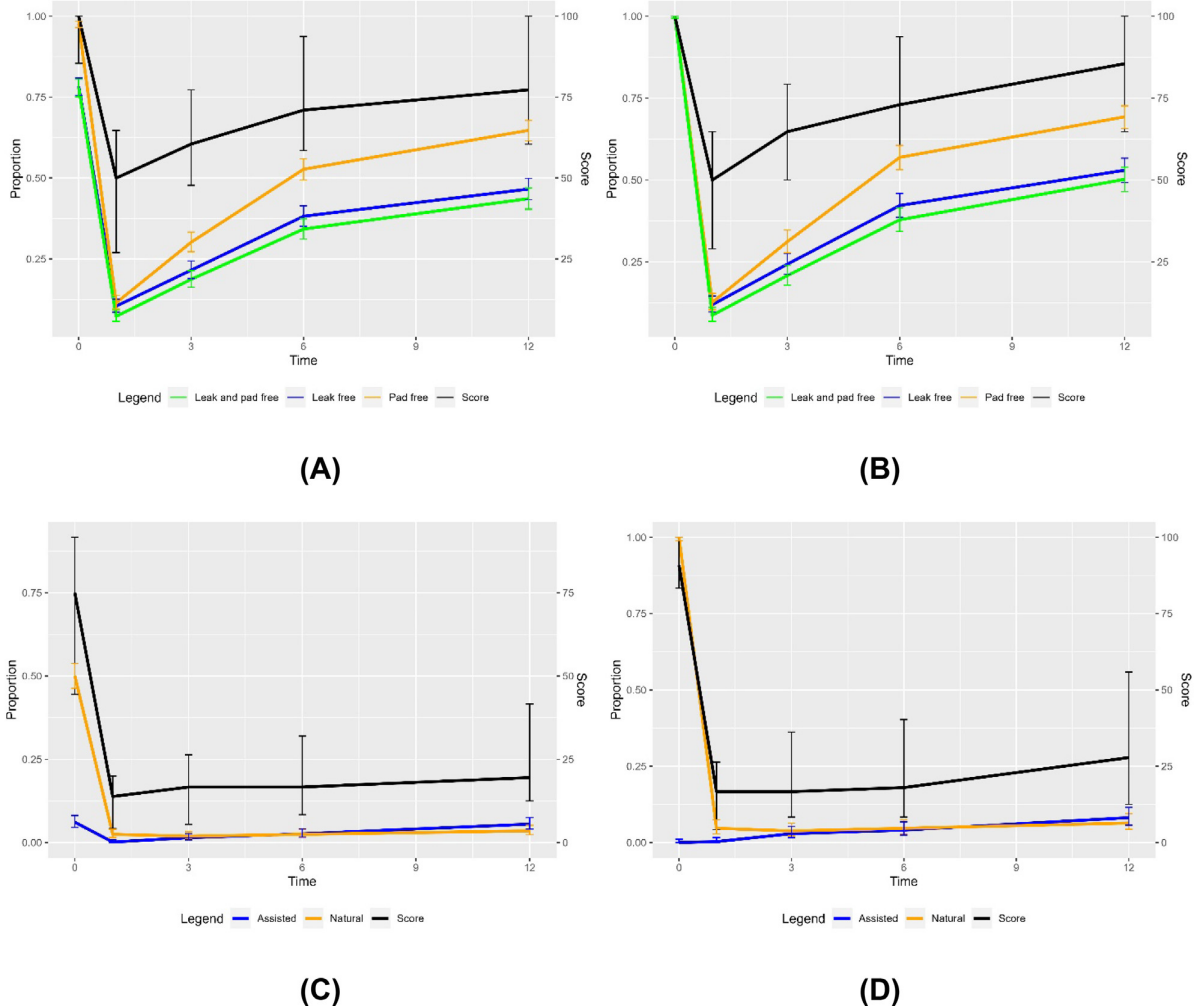
Recovery of urinary and sexual function over the first year after radical prostatectomy among men with patient-reported outcome measure data at each time point. Urinary function for (A) all men and (B) men who were leak-free and pad-free at baseline. Sexual function for (C) all men and (D) men with erections sufficient for intercourse at baseline.

**Fig. 2:**
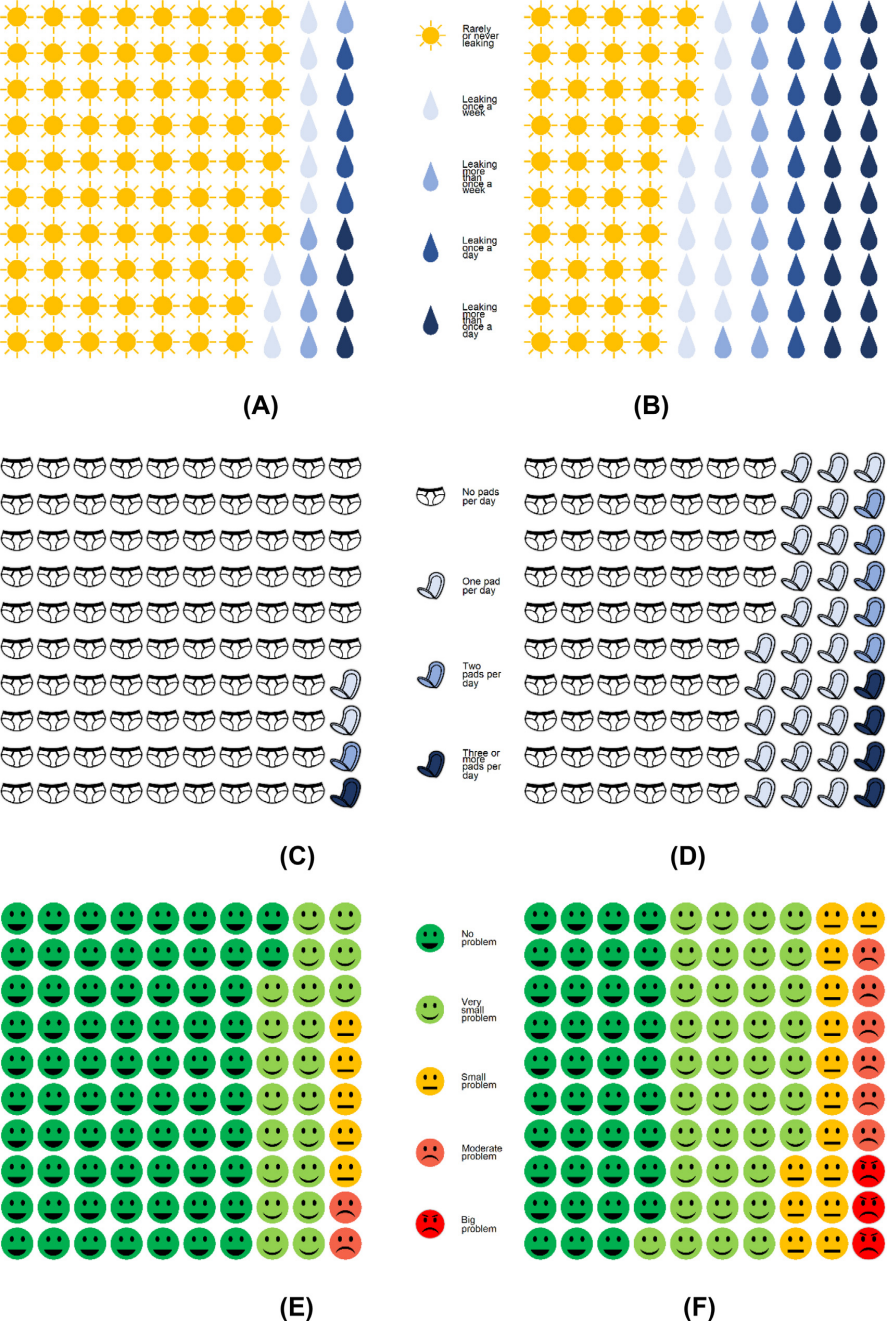
Urinary leakage at (A) baseline and (B) 12 mo. Pad use at (C) baseline and (D) 12 mo. Urinary leakage bother at (E) baseline and (F) 12 mo.

Urinary function recovery over 12 mo is shown in Figure 1. At baseline, 3% of men reported any pad use, (68/1996; 95% CI 3–4%) with 43 men (2%) using 1 pad/d, 11 men (1%) using 2 pads/d, and 14 (1%) using >2 pads/d (Fig. 2). At baseline, one in four men reported any leakage (460/1996; 23%, 95% CI 22–25%) The median EPIC-26 score for these 1996 men at baseline was 100 (IQR 86–100; Fig. 1 and Table 3).

Of 1388 men who provided urinary PROM data at 12 mo, 65% were pad-free (904/1388; 95% CI 63–68%) and 42% were pad-free and leak-free (583/1388; 95% CI 39–45%). The majority of those who wore pads wore 1 pad/d (363, 26%), with 68/1338 (5%) wearing 2 pads/d and 53/1338 (4%) wearing >2 pads/d (Fig. 2). The median EPIC-26 incontinence domain score among these 1388 men was 76 (IQR 61–100; Fig. 1 and Table 3).

Supplementary Figures 2 and 3 show parallel coordinate plots demonstrating the change in leakage and pad use for each man between baseline and 12 mo.

#### 3.1.1 Subgroup analysis of men with no leakage or pad-use at baseline

From the subgroup of 1536 men who were pad-free and leak-free at baseline, 1086 reported 12-mo outcomes (Supplementary Fig. 4). Of these, 70% (758/1086; 95% CI 67–72%) remained pad-free at 12 mo, with 48% remaining pad-free and leak-free (523/1086; 95% CI 45–51%). The median EPIC-26 incontinence domain score was 84 (IQR 65–100; Table 3).

#### 3.1.2 How often do men who wear pads leak urine?

Most men (≥90%) wearing pads experienced a leak at least once a week, with one in five men at baseline and one in three men at 12 mo leaking urine once a day, and not wearing a pad (Supplementary Fig. 5).

#### 3.1.3 How much of a problem is urine leakage and pad-use in the first year after RP?

At baseline, 18 men (2%, 95% CI 1–3%) reported a moderate or big problem with urine leakage. Despite the fact that one in three men were wearing pads and one in two men experienced urine leakage, at 12 mo after RP only 89 men (10%, 95% CI 8–12%) reported a moderate or big problem with urine leakage or dripping (Fig. 2).

The magnitude of the problem associated with urine leakage increases with the frequency of leakage and the wearing of pads, with most men who reported a big problem leaking more than once a day and wearing ≥2pads/d (Supplementary Figs. 6 and 7). One in four men (77/326; 24%) who reported wearing at least 1 pad/d reported a moderate or big problem with urine leakage (Supplementary Fig. 7).

### 3.2 Analysis of sexual function recovery

Recovery of sexual function over the first year after RP is shown in Figure 1.

Among 1200 men who reported sexual function PROM data at baseline and 12 mo (Table 3), 4% reported natural erections adequate for sexual intercourse at 12 mo (42/1200; 95% CI 3–5%), with an additional 7% reporting erections adequate for sexual intercourse with tablets or medical assistance (83/1384; 95% CI 5–8%) and 90% having no adequate erections (1075/1200; 95% CI 88–91%; Supplementary Fig. 9). The median EPIC-26 sexual domain score at 12 mo among 1200 men was 18 (IQR 8–40; Fig. 1 and Table 3).

Supplementary Fig. 8 shows a parallel coordinate plot demonstrating the change in erection firmness for each man between baseline and 12 mo.

#### 3.2.1 Subset analysis of men who had natural erections firm enough for intercourse at baseline

**Fig. 3:**
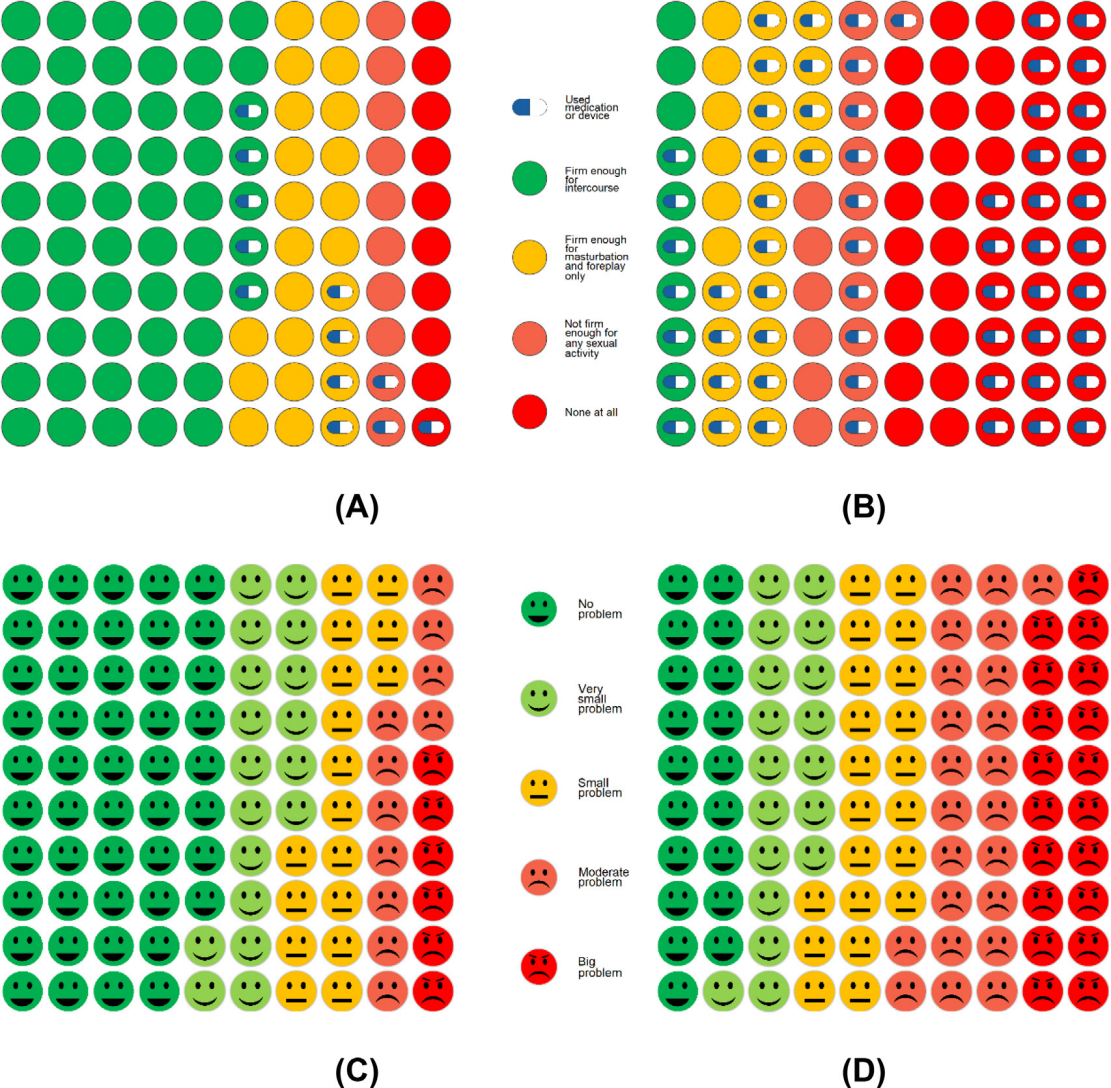
Erection quality and use of medication or devices at (A) baseline and (B) 12 mo. Sexual function bother at (C) baseline and (D) 12 mo.

At baseline, 975/1880 men (52%, 95% CI 50–54%) reported unassisted natural erections firm enough for intercourse (Fig. 1). Among these 975 men, 630 reported 12-mo data, with 37/630 (6%, 95% CI 4–8%) continuing to have natural erections firm enough for intercourse and an additional 11% (68/630; 95% CI 9–13%) having assisted erections firm enough for intercourse (Fig. 3), while 83% had no adequate erections at 12 mo (525/630; 95% CI 80–86%; Fig. 1). The median EPIC-26 sexual domain score was 33 (IQR 21–60; Fig. 1 and Table 3).

There were 630 men with natural erections at baseline who had completed 12-mo data, including the use of sexual medicines. Among these men, 432 had tried pills, of whom 181/432 (42%) found medication helpful at some point and 84/432 (20%) were still finding medication helpful at 12 mo. A minority of men (21/630, 3%) had tried an intraurethral pellet, but only 8/21 (38%) had found this helpful. A small number of men (45/630, 7%) had tried an intracavernosal injection, with the majority 32/45 (71%) finding this option helpful. Vacuum pump use was more common, with 300/630 men (48%) having tried a vacuum pump at some time point, and the majority (230/300, 77%) finding it helpful, while 73/300 (24%) had tried a pump but were no longer using it by 12 mo.

## 4 Discussion

### 4.1 Summary of results

In a cohort of 2030 men, one in three reported wearing of pads for urine leakage and one in two reported either urine leakage or wearing of pads at 12 mo after RP. The majority of pad-wearing men reported use of only 1 pad/d, and leaking urine at least once per week. At 12 mo, only one in ten men reported urine leakage as a moderate or big problem. Of the men who had erections without medication or device assistance at baseline, one in 20 maintained erections without medication, and one in ten had erections sufficient for intercourse with medication.

### 4.2 Comparison of urinary function to other cohorts

As EPIC-26 domain scores are highly skewed, we consider that median values are more appropriate than mean values; however, as previous studies have reported mean values, we calculated mean values for comparison, and report these below.

The TrueNTH Post Surgery study took place at a similar time to PROM data collection in the UK National Prostate Cancer Audit (NPCA) [5]. The NPCA used a paper approach for data collection (compared to electronic PROMs in TrueNTH) and had a response rate of approximately 75% at a single time point. The NPCA used cancer registry data to identify patients, with PROM data collection at 12–18 mo after diagnosis, which may be vary from the date of surgery; by contrast, our approach used time from the date of surgery and collected data at baseline and at 1, 3, 6, and 12 mo after surgery.

Nossiter et al [5] reported data for 7702 NPCA responders who underwent RP between April 2014 and September 2016, and found that higher-volume centres were associated with slightly higher scores. Mean incontinence scores ranged from 69.5 to 72.6, in comparison to 75.8 in our cohort.

We know that prostate cancer risk categorisation can impact functional outcomes, as it can influence decisions such as the degree of nerve sparing. In the NPCA cohort, 8.9% of men had Gleason 6 disease [6], compared to 7.8% of men in TrueNTH. However, if low risk is defined according to D’Amico risk groups (Gleason score 6, prostate-specific antigen <10 ng/ml, and clinical stage ≥T2a [confined to less than half of one lobe]), 4.2% of men in TrueNTH and 0.7% of men in the NPCA analysis would meet these criteria, which could account for the slightly higher functional scores in TrueNTH Post Surgery.

It is possible that surgeons in TrueNTH were self-selected and working at higher-volume centres than the broader NPCA population. However, the participants in TrueNTH were a subset of the invited population and may represent those with other characteristics that might predispose to better recovery (eg, younger age).

Recovery may well continue past the first year, so our 12-mo time point might underestimate the full extent of recovery. A single-centre analysis for more than 1000 men from East Kent Hospitals showed a pad-free rate of 79% at 1 yr (compared to 65% in TrueNTH), which increased to 83% at 18 mo and 85% at 24 mo [7].

The UK Life After Prostate Cancer Diagnosis (LAPCD) study was also running in the UK at the same time as TrueNTH Post Surgery, but was designed to capture those not in the NPCA PROM analysis [8], [9]. The LAPCD study sampled 35 000 men across England, Scotland, Wales, and Northern Ireland, among whom 7000 patients had surgery alone. EPIC-26 surveys were conducted 18–42 mo after diagnosis. For the men who underwent surgery alone, mean EPIC-26 scores were 73.5 (95% CI 72.8–74.1) for the urinary incontinence domain and 22.1 (95% CI 21.5–22.6) for the sexual function domain, similar to results in our cohort.

The UK PROTECT study assessed men who were randomised between active monitoring, open RP, and radical radiotherapy, and has robust follow up data over several years [10]. Of the 750 men who underwent RP, 36% were wearing pads at 1 yr, which decreased to 20% by 6 yr. The pad rate at 1 yr is very similar to the 35% in TrueNTH.

LAP-01 was a randomised study of robotic versus laparoscopic RP (3:1 randomisation) [11]. Among men who had robotic surgery, urine leakage was reported as more than once a week by 32% at 12 mo, in comparison to 41% in TrueNTH.

A large surgical quality improvement collaborative in Michigan reported a mean EPIC-26 urinary function score of 73.7 at 12 mo, similar to the mean score of 73.8 in the UK NPCA, and 75.8 in TrueNTH [12]. The Michigan group showed significant variability in “good” urinary function (EPIC-26 score ≥74) across surgeons, ranging between 0% and 54.5%.

The disease characteristics, comorbidities, prior functional status, and age of men undergoing RP are all expected to have a significant impact on functional outcomes. This TrueNTH Post Surgery cohort has a lower proportion of men with low-risk disease (48/929, 5.2%) in comparison to many US national cohorts (eg, 43% and 45%), reflecting the fact that only 6.4% of men in the UK with low-risk disease undergo surgery, in comparison to 22% in the USA.

Following UK national guidance published in 2002, RP has been concentrated in centres performing at least 50 procedures per year. The NPCA data show that out of 49 surgical centres across England and Wales, only one had a significantly worse score for incontinence than the national average [13].

### 4.3 Comparison of sexual function outcomes to other cohorts

In a single-institution study by Walsh et al [14] in a small, well-selected group of men with low-risk disease, more than 80% of men had erections sufficient for intercourse after surgery, with one-third using phosphodiesterase inhibitors to achieve erection. It is notable that most men (87.5%) in the study by Walsh et al, which was published in 2000, had Gleason-6 disease, compared to 7.8% in our cohort, and 89% underwent bilateral nerve-sparing RP, compared to 39% in our cohort. Additional factors to explain these higher rates of recovery could be the longer time for recovery and differences in the patient population at baseline, including disease stage. Thus, while exceptional results might be achieved in young and healthy men with low-risk disease undergoing meticulous nerve-sparing surgery in selected centres, these results are not comparable to contemporary UK practice, which involves a far greater proportion of cases of intermediate- and high-risk disease, and an older population.

A study by Sanda et al [15] involved a cohort of 603 men who underwent RP; EPIC-26 sexual function domain scores reported by 557 of these men at 12 mo ranged from 20 to 40, depending on the nerve-sparing approach, with some further improvement by 24 mo only in those who had nerve-sparing surgery. Results for the same cohort at 24 mo were reported by Alemozaffar et al [16]: 40% of men had erections sufficient for penetration, with two in three of these men needing medication or a device, in comparison compared to >20% of men having erections sufficient for penetration at 12 mo, showing the potential for further recovery after 12 mo.

Mean NPCA sexual function scores ranged from 18.7 to 26.6 [5] in comparison to 34.7 in our cohort.

### 4.4 Limitations

Our study may be subject to reporting bias because men who choose to report functional outcomes after surgery may be more likely to do so if they have serious side effects. The use of an online data collection tool (in contrast to the UK NPCA approach of postal questionnaires) prompted concern that younger men took part in comparison to the NPCA data set. However, men who reported functional outcomes did not differ in clinical or demographic characteristics from men who did not report those outcomes in our study.

The NPCA reported that between April 2015 and March 2019, 15 480 RP procedures were performed at the centres in our study. Our sample of 2030 cases represents only 13% of the RPs performed in these centres around the same time.

Juxtaposition of EPIC-26 domain scores to specific patient-reported information on urinary continence and potency means that both clinicians and men with prostate cancer can be better informed about expected outcomes and recovery.

We acknowledge that nerve-sparing details can be reported in different ways. After discussion at the TrueNTH UK surgical forum before data collection, it was decided to record nerve-sparing details in a simple manner indicating unilateral, bilateral, or no nerve sparing to maximise the likelihood of data capture.

The use of preoperative and postoperative rehabilitation approaches, including pelvic floor rehabilitation and routine use of PDE5 inhibitors, was not formally reported in this study, and dedicated strategies for rehabilitation were not common at the time of patient recruitment. Addressing the role of rehabilitation would offer potential to improve the functional outcomes reported here.

## 5 Conclusions

These results provide both EPIC-26 domain scores and descriptive terms for urinary and sexual function, along with problem scores in the first year after RP, from a large multicentre collaboration in the UK. The data show that RP in the UK, as currently used for intermediate- and higher-risk disease, affects quality of life, with a greater effect on sexual function in comparison to urinary function.

While the urinary and sexual function domain scores in this study roughly mirror large multicentre studies in the literature, the use of clinical outcome information reported by patients (eg, urine leakage, pad use, ability to achieve erections with and without tablets) alongside EPIC-26 domain scores gives a more readily understandable assessment of how quality of life is affected, which both clinicians and patients should be aware of when considering decisions about prostate cancer treatments for localised disease.

  ***Author contributions***: Caroline M. Moore had full access to all the data in the study and takes responsibility for the integrity of the data and the accuracy of the data analysis.

  *Study concept and design*: Moore, Smith, Protopapa, van der Meulen.

*Acquisition of data*: Moore, Hamer, McCartan, Brew-Graves.

*Analysis and interpretation of data*: Moore, Bridge, Mallett, Cole, Trinh, van der Meulen.

*Drafting of the manuscript*: Moore, Adebusoye, Cole, Bridge, Mallett.

*Critical revision of the manuscript for important intellectual content*: Cole, Trinh, van der Meulen, Bridge, Labban.

*Statistical analysis*: Bridge, Mallett.

*Obtaining funding*: Moore.

*Administrative, technical, or material support*: McCartan, Brew-Graves, Hamer.

*Supervision*: Moore, Mallett, van der Meulen.

*Other*: None.

  ***Financial disclosures:*** Caroline M. Moore certifies that all conflicts of interest, including specific financial interests and relationships and affiliations relevant to the subject matter or materials discussed in the manuscript (eg, employment/affiliation, grants or funding, consultancies, honoraria, stock ownership or options, expert testimony, royalties, or patents filed, received, or pending), are the following: Quoc-Dien Trinh reports personal fees from Astellas, Bayer, and Janssen outside the submitted work. Caroline M. Moore reports speaker fees from Astellas and Janssen, and proctoring fees from Sonablate. The remaining authors have nothing to disclose.

  ***Funding/Support and role of the sponsor*:** Prostate Cancer UK funded TrueNTH as part of the Movember TrueNTH initiative. The sponsor played no direct role in this study.

  ***Acknowledgments*:** Alexander P. Cole and Quoc-Dien Trinh are supported by a grant from the American Cancer Society and Pfizer Global Medical Grants. Caroline M. Moore is an NIHR Research Professor and receives grant funding from the Medical Research Council, Cancer Research UK, Movember, Prostate Cancer UK, and the EAU Foundation, and study funding from SpectraCure. Jan Van Der Meulen is a member of the National Prostate Cancer Audit project team.

  ***Data sharing statement*:** The data owner is Caroline M. Moore. The data are held at University College London, and requests for anonymised subsets of data can be submitted 3 yr after publication.

## Appendix A True NTH post surgery UK investigators

Vanessa Adamson, Leighton Hospital, Mid Cheshire Hospitals NHS Trust; Jim Adshead, Tim Lane, and Nikhil Vasdev, Hertfordshire and Bedfordshire Urological Cancer Centre; Chris Anderson, Rami Issa, and Hasan Qazi, St George’s Hospital, London; Jonathan Aning, Freeman Hospital, Newcastle, and Bristol Urological Institute; Tim Briggs, My Medical Records, University Hospital Southampton; Christian Brown, Paul Cathcart, Ben Challacombe, Prokar Dasgupta, Rajesh Nair, and Rick Popert, Kings College London & Kings Health Partners; Declan Cahill, Urology Unit, Royal Marsden NHS Foundation Trust;, Isobelle Coombes, Royal Sussex County Hospital, Brighton; Tim Dudderidge and John Dyer, University Hospital Southampton NHS Foundation Trust; Steve Garnett, East Sussex Healthcare NHS Trust; Chris Hough, Mid Cheshire Hospitals NHS Foundation Trust; Kay Jones and Matin Sherif, Medway Hospital, Gillingham; Mark Johnson and Naem Soomro, Freeman Hospital, Newcastle; Anand Ramesh Kelkar, Muthiah Senthil Nathan, Prabhakar Rajan, Gregory Shaw, and Prasanna Sooriakumaran, University College London Hospitals Trust; Anthony Koupparis, Raj Persad, and Edward Rowe, Bristol Urological Institute; Marc Laniado, Royal Berkshire NHS Trust; Maurice Wai-Ming Lau and Vijay Ramani, The Christie Hospital, Manchester; Naing Lynn, Shrewsbury & Telford NHS Trust; Alan McNeill, Western General Hospital, Edinburgh; Salvatore Natale, Derriford Hospital, Plymouth; Matthew Perry, Royal Surrey County Hospital, Guildford; Sally Potts, Shrewsbury and Telford Hospital; Stephanie Ridgeway, Tameside General Hospital, Ashton under Lyne; Nimesh Shah, Addenbrookes Hospital, Cambridge; Plavai Sreekanth, Southend Hospital; Ashwin Narasimha Sridhar, My Medical Records, University Hospital Southampton; Shalom Srirangam, Royal Blackburn Hospital; Kevin Turner and Andrew Wedderburn, Royal Bournemouth Hospital; Linda Wagstaff, Walsall Manor Hospital; Ann Wearing, Preston County Hospital; and Simon Williams, University Hospitals of Derby and Burton NHS Foundation Trust.
