# Supplementary material for: Urinary and Sexual Impact of Robotic Radical Prostatectomy: Reporting of Patient-reported Outcome Measures in the First Year after Radical Prostatectomy in a Contemporary Multicentre Cohort in the United Kingdom
Source: Eur Urol Open Sci. 2024 May 21;64:11–21. doi: 10.1016/j.euros.2024.05.003 (PMC11134924; doi:10.1016/j.euros.2024.05.003)
Supplement: Supplementary Data 1 [file mmc1.pdf]

Supplementary material

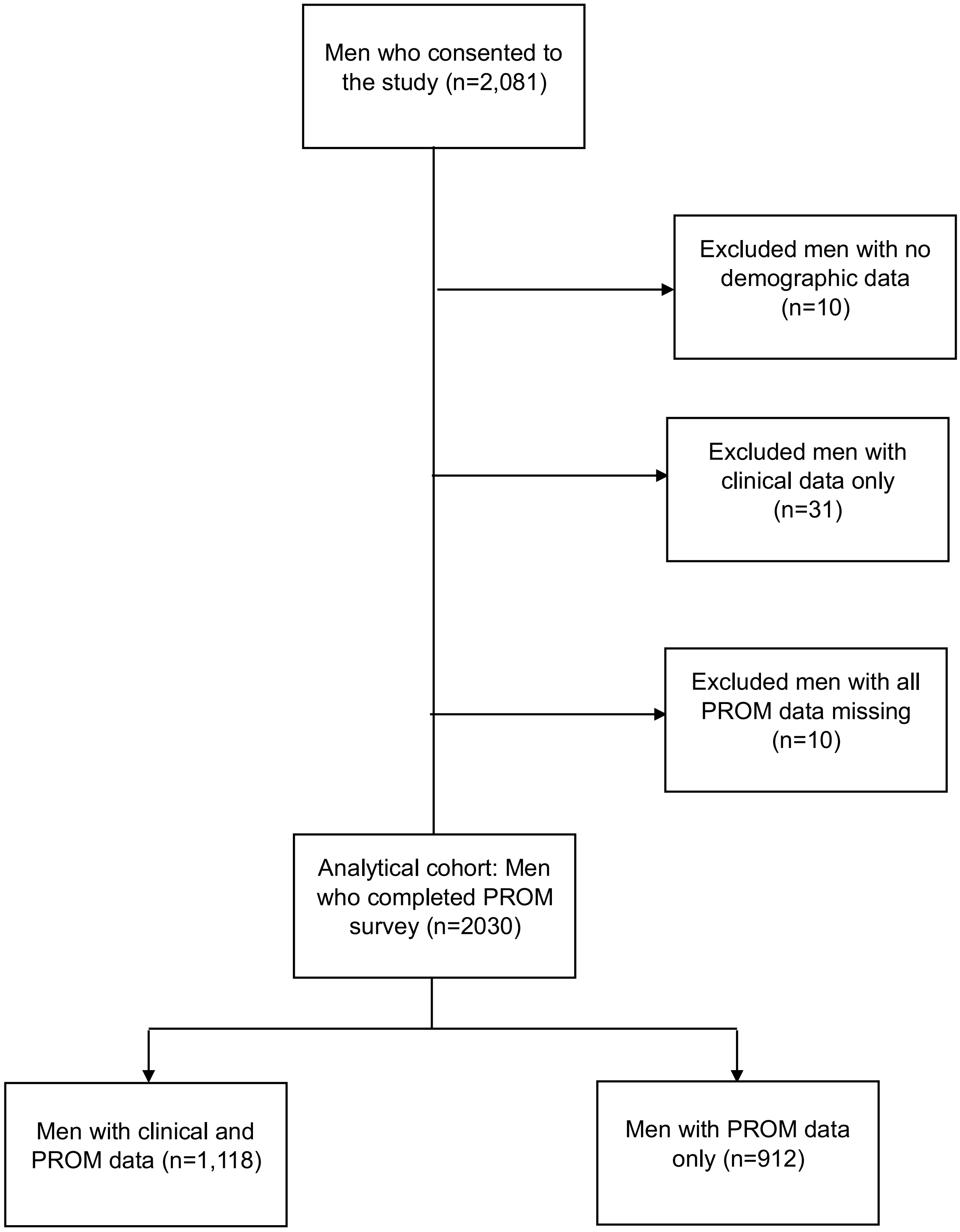

Supplementary Fig. 1: Consort Diagram for patients who were recruited through TrueNTH Post Surgery UK who underwent radical prostatectomy between November 2015 and September 2019.

Additional figures for urinary function

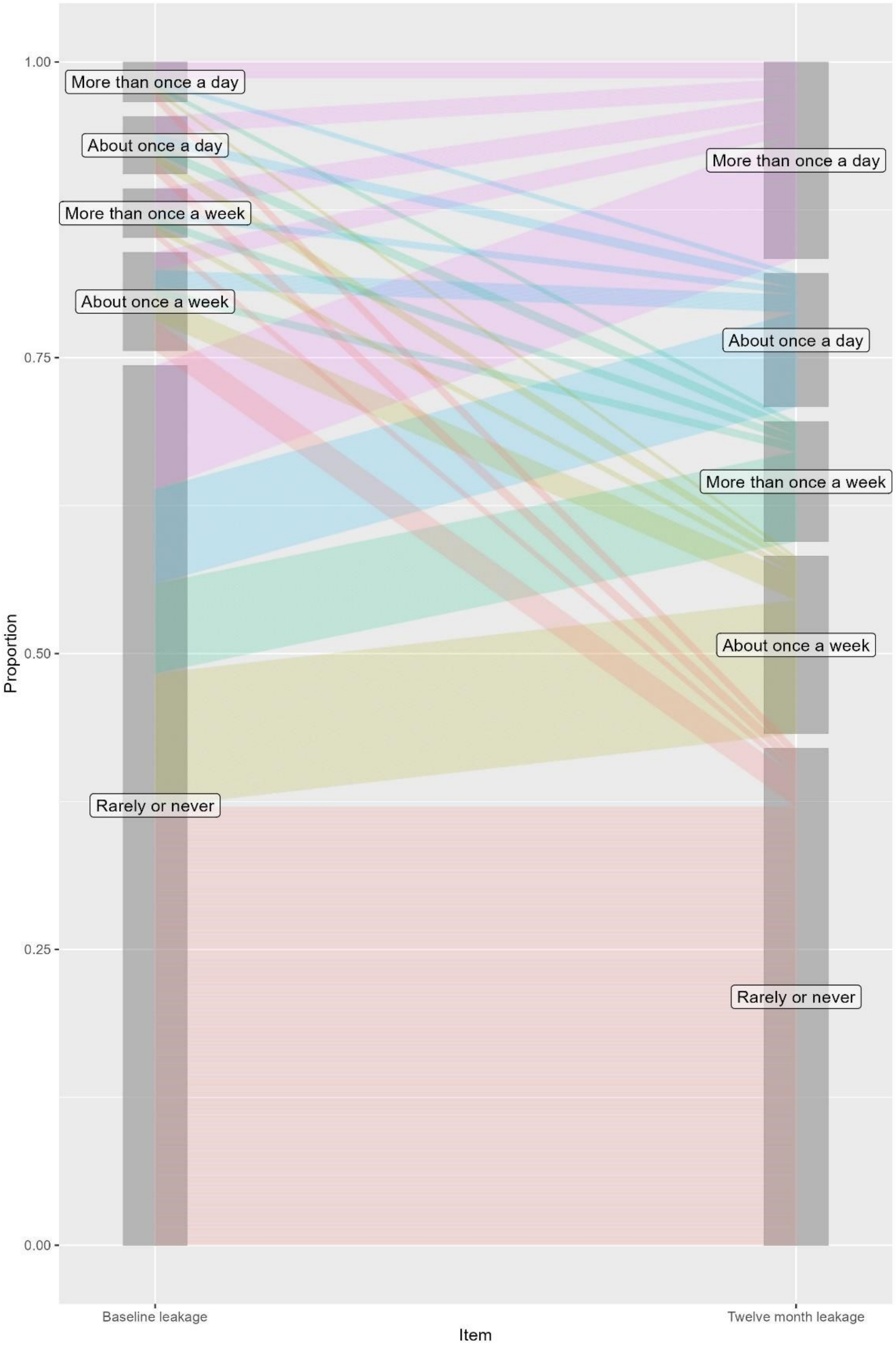

Supplementary Fig. 2: Parallel coordinate plot showing the number of men with leakage at baseline and 12-months and their progression between these two time points. The colour corresponds to the 12-month outcome.

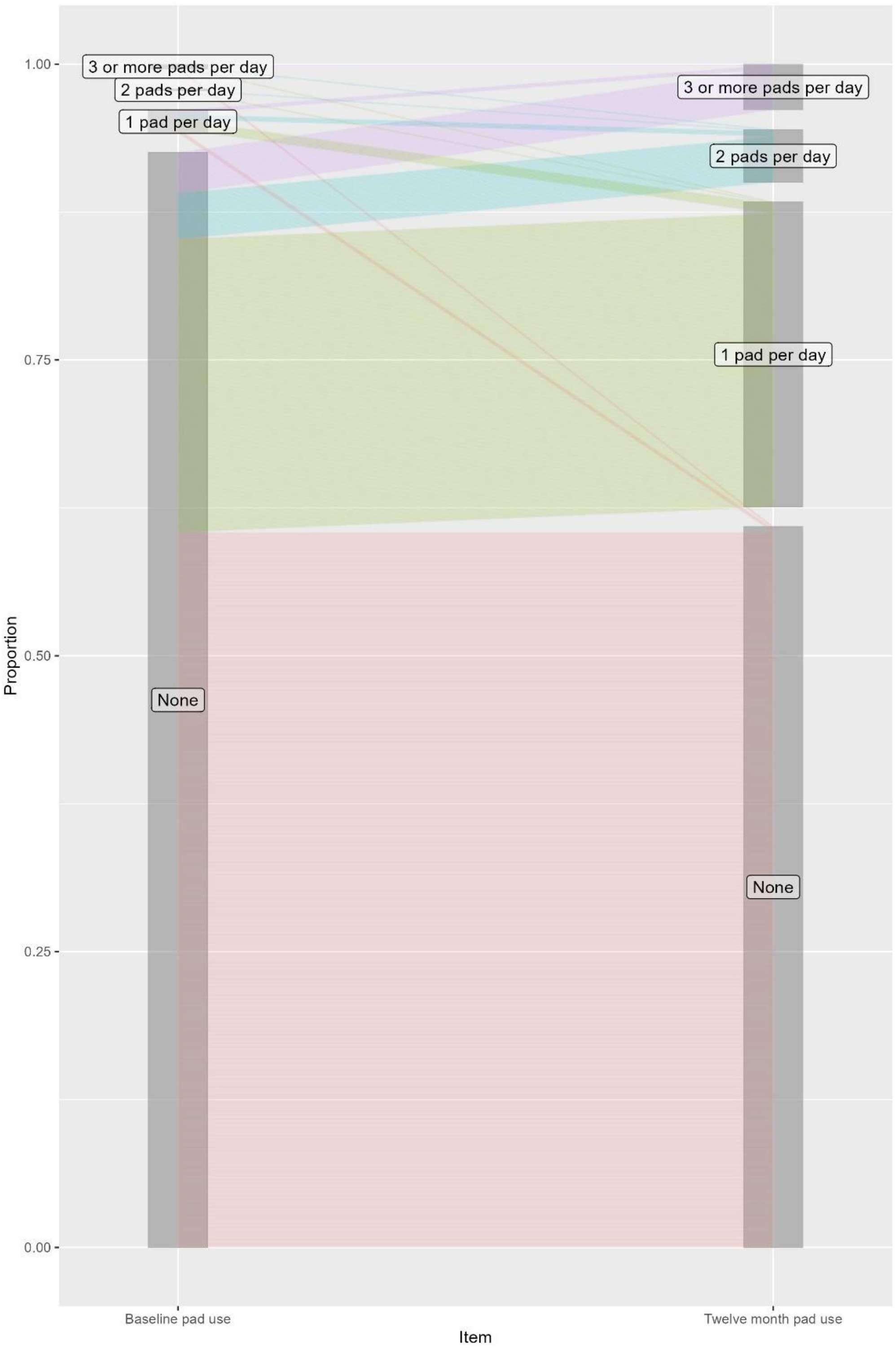

Supplementary Fig. 3: Parallel coordinate plot showing the pad use at baseline and 12-months and their progression between these two time points. The colour corresponds to the 12-month outcome.

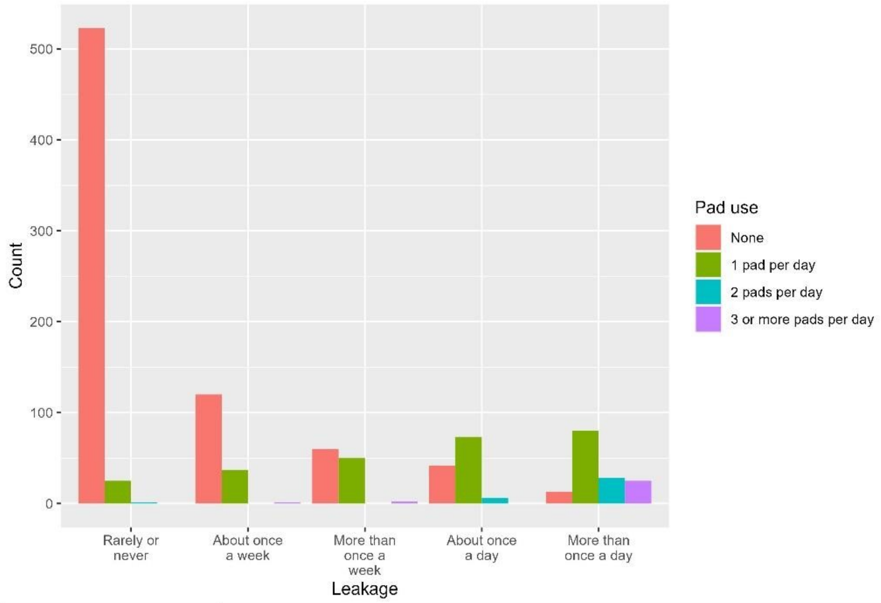

Leakage	Pad use				
	None	1 pad per day	2 pads per day	3 or more pads per day	Total
Rarely or never	523	25	1	0	549
About once a week	120	37	0	1	158
More than once a week	60	50	0	2	112
About once a day	42	73	6	0	121
More than once a day	13	80	28	25	146
Total	758	265	35	28	1,086

Supplementary Fig. 4: Leakage and pad use at 12 months for those men who were leak free and pad free at baseline

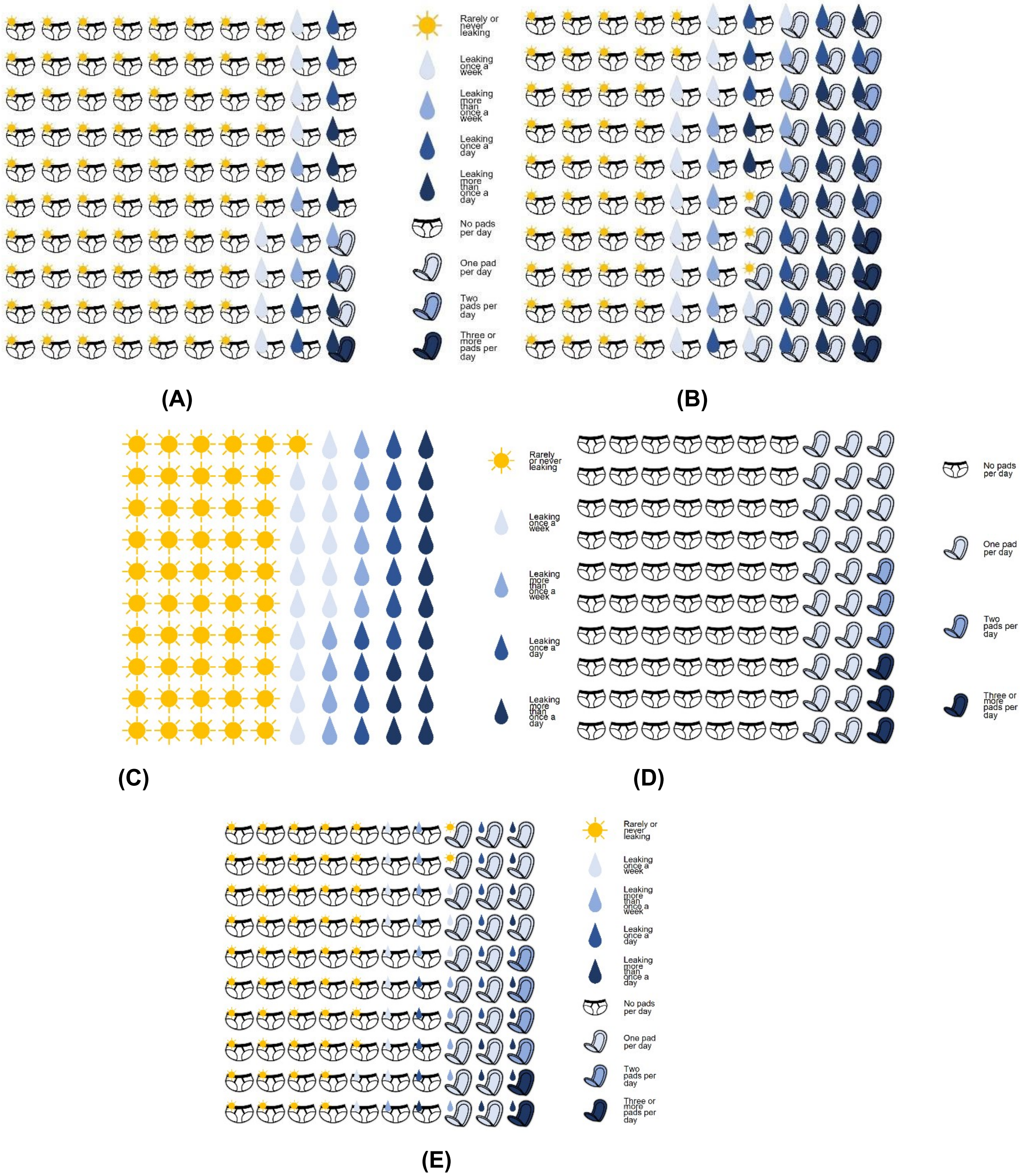

Supplementary Fig. 5: Urine leakage and pad use over the past 4 weeks at (A) Baseline vs (B) 12 months for all patients. For patients who were leak-free and pad-free at baseline (C) leakage, (D) pad use, and (E) leakage and pad use.

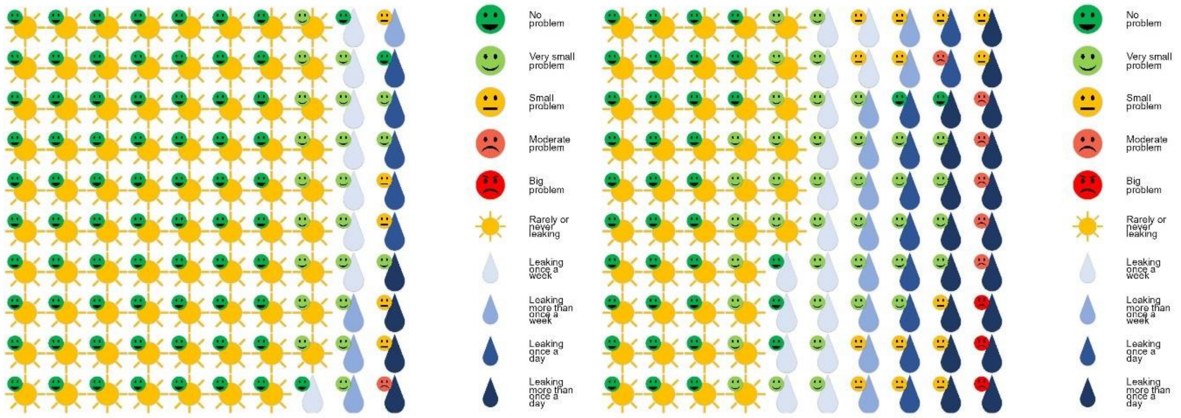

(A) (B)

Supplementary Fig. 6 Urine leakage and problems with urine leakage over the past 4 weeks at (A) Baseline and (B) 12 months.

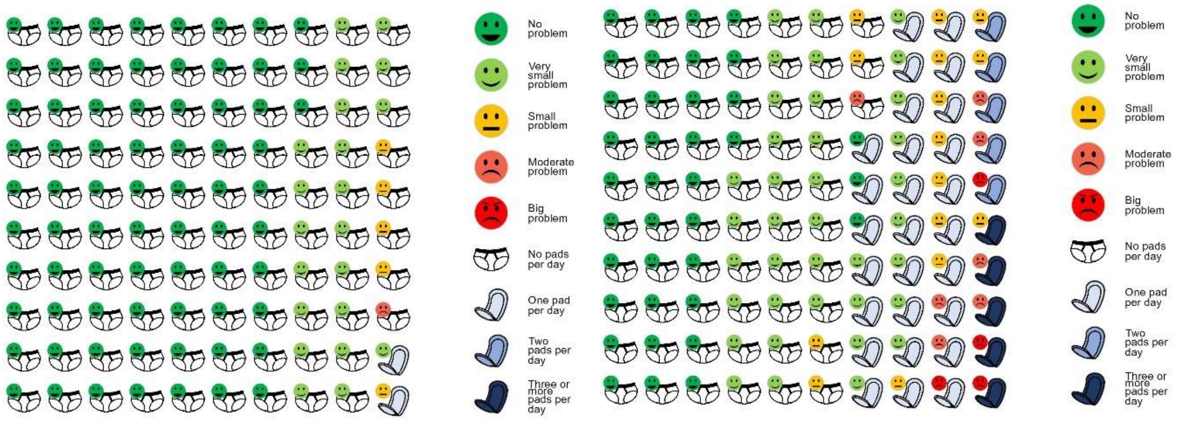

(A) (B)

Supplementary Fig. 7: Pad use and problems with urine leaking or dripping over the past 4 weeks at (A) Baseline and (B) 12 months

Additional figures for sexual function

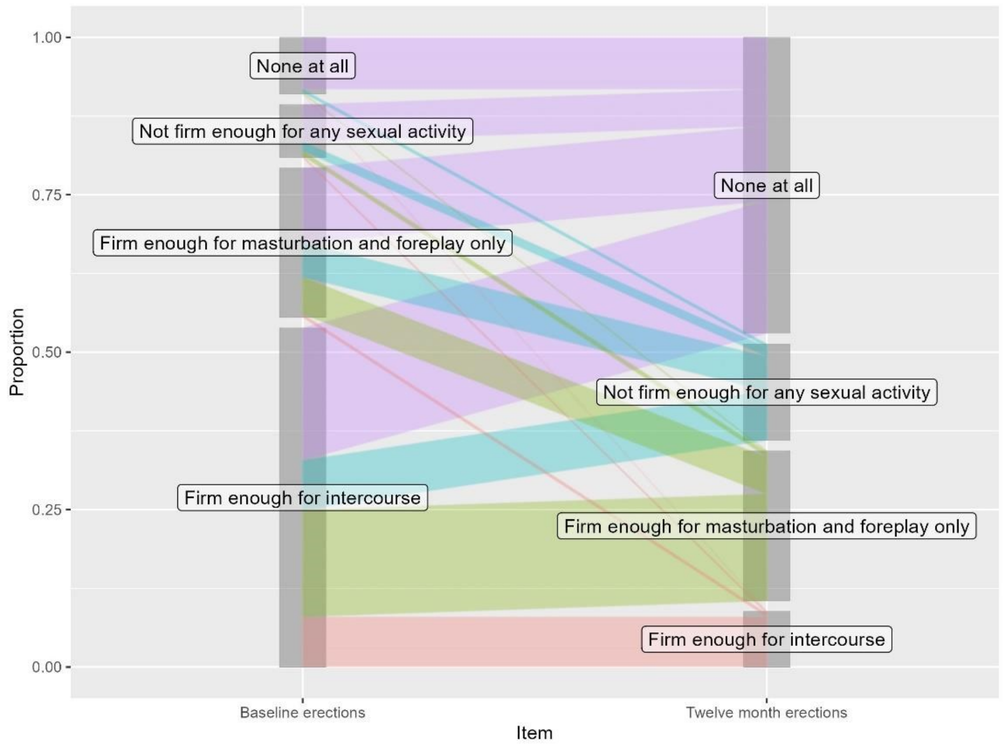

Supplementary Fig. 8: Parallel coordinate plot showing erection quality at baseline and 12-months and their progression between these two time points. The colour corresponds to the 12-month outcome.

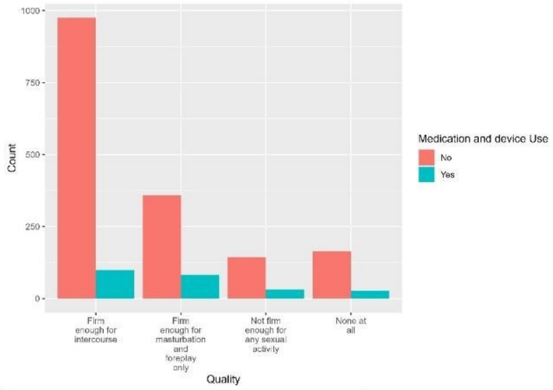

Quality	Medication and device Use		
	No	Yes	Total
Firm enough for intercourse	975	99	1,074
Firm enough for masturbation and foreplay only	358	82	440
Not firm enough for any sexual activity	144	31	175
None at all	164	27	191
Total	1,641	239	1,880

(A)

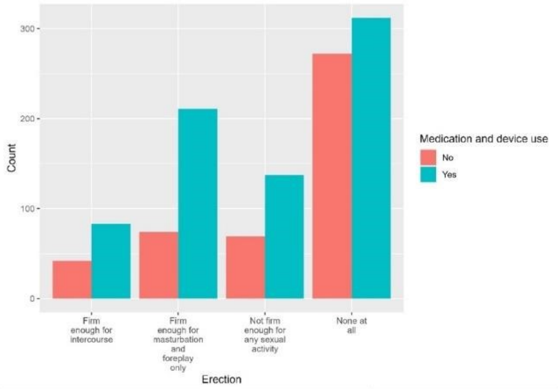

Erection	Medication and device use		
	No	Yes	Total
Firm enough for intercourse	42	83	125
Firm enough for masturbation and foreplay only	74	211	285
Not firm enough for any sexual activity	69	137	206
None at all	272	312	584
Total	457	743	1,200

(B)

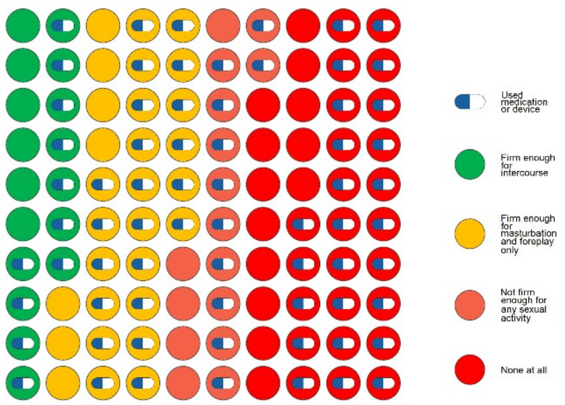

(C)

Supplementary Fig. 9: Erection firmness and use of medication or devices at (A) baseline and (B) 12 months. Waffles plots for patients with baseline erections firm enough for intercourse without using medication or devices showing (C) erection quality and medication or device use.

Multiple imputation

Any patient reported data are likely to have missing values. Multiple imputation was previously proposed as a method of handling missing PROM data.[1] The percentages of missing data for age, urinary incontinence and sexual function domain responses, and assistance use at each time point are shown in Table SE1. All responses are needed for the urinary incontinence score; however, for the sexual function domain score, one missing response is allowed.[2] The percentage of missing urinary incontinence domain and sexual function domain scores is shown in Table SE2.

No patients had responses missing at all time points; therefore, the missing outcomes are likely to depend only on the observed variables and not the unobserved variables. At each follow-up point, the percentages of missing data remain stable. There is no pattern to the missing data when considering all variables, although sexual function outcomes show slightly more missingness than urinary incontinence outcomes.

As a sensitivity analysis, we imputed missing data to compare with the complete case results. We imputed the PROMs which are used for the urinary incontinence and sexual domains, along with use of medications or devices, and age, across all time points; around 25% of this information was missing.

For imputation, we used multiple imputation with chained equations, implemented in the mice package in R.[3] This creates multiple possible imputed datasets which can then be combined to obtain estimates of outcomes. The imputation dataset included age at surgery, the responses used to calculate the urinary incontinence and sexual function domain scores at

each time point, and whether erection assistance (e.g., pills or devices) were used at each time point. We then imputed the missing data at each time point within a single multiple imputation.

We imputed 25 datasets. The EPIC-26 questions were treated as ordinal data, the use of medication for erections was treated as binary, and age was treated as continuous. Age was observed to be approximately normally distributed and needed no transformation.

For the proportions we calculated the pooled proportion and constructed the confidence intervals using Wilson intervals. This was performed using the *pool_prop_wilson* function in the R package miceafter.[4]

Visual comparisons show pooled point estimates of proportions for patients with complete data and the imputed data containing all patients; 95% confidence intervals are constructed using the Wilson method. We found that results are similar between the complete case data and the imputed data.

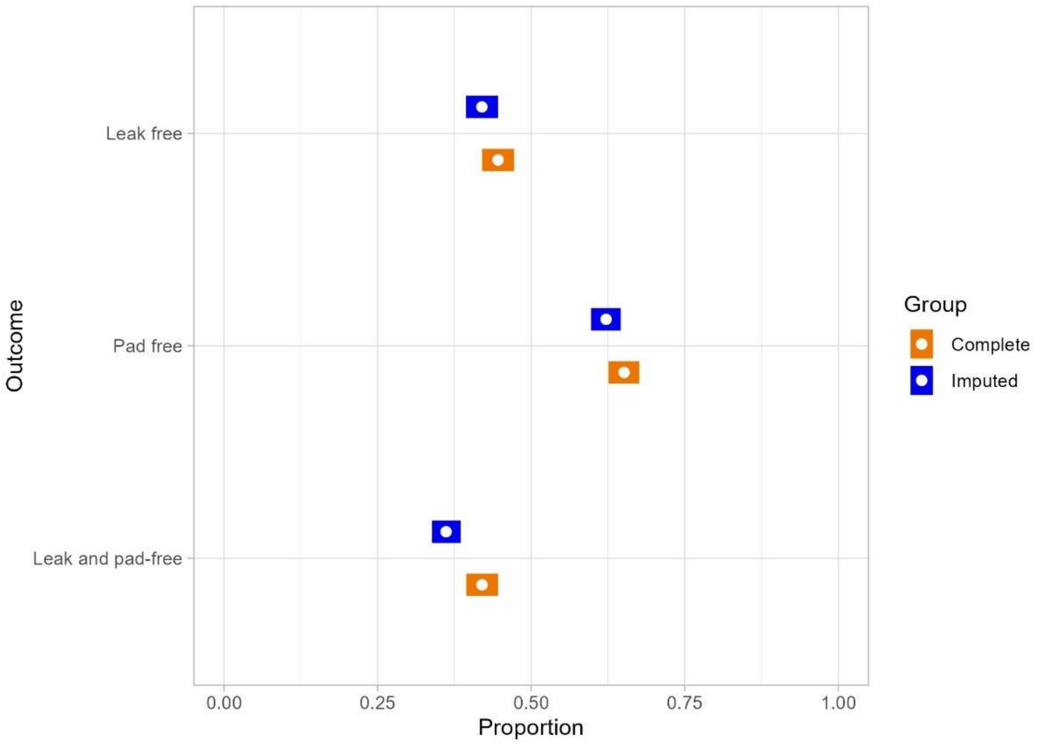

Supplementary Fig. 10: Plot showing the differences between point estimates and 95% confidence intervals in complete case and imputed data for urinary outcomes for all patients.

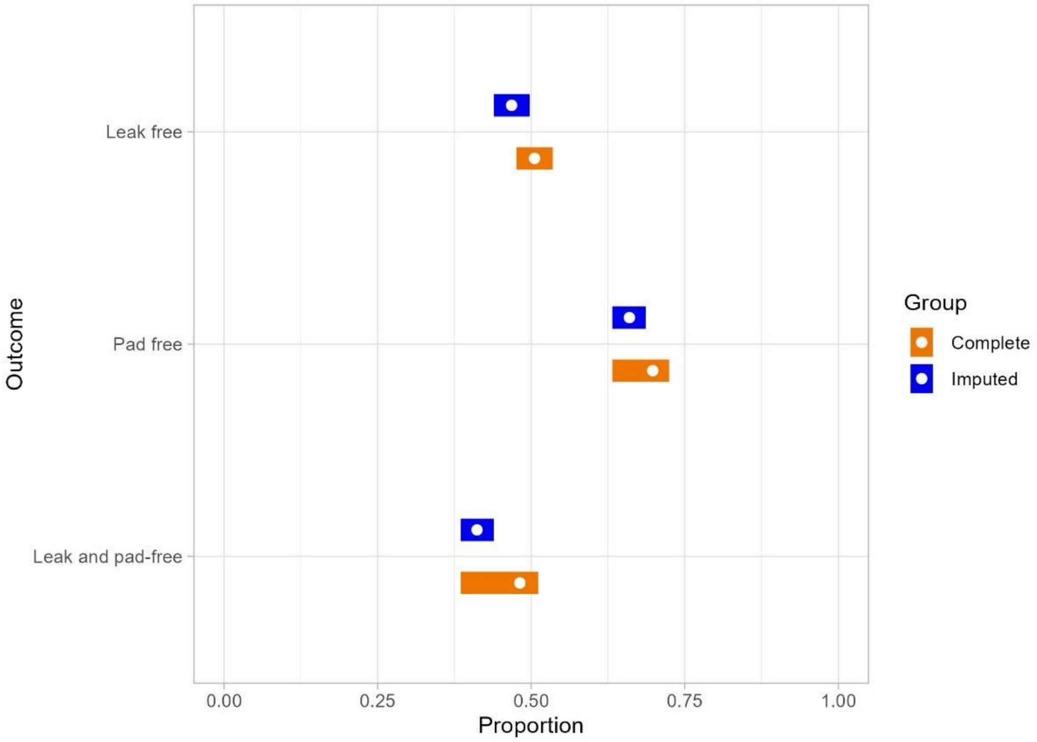

Supplementary Fig. 11: Plot showing the differences between point estimates and 95% confidence intervals in complete case and imputed data for urinary outcomes for patients who were leak free and pad free at baseline.

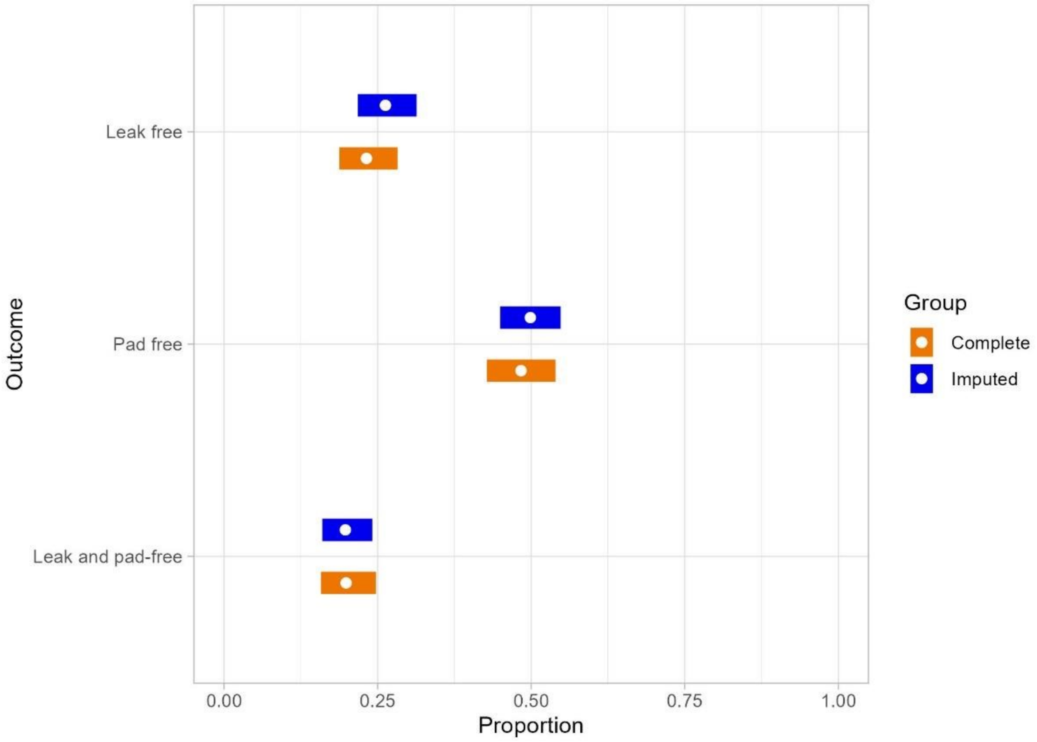

Supplementary Fig. 12 Plot showing the differences between point estimates and 95% confidence intervals in complete case and imputed data for urinary outcomes for patients who were not leak free and pad free at baseline.

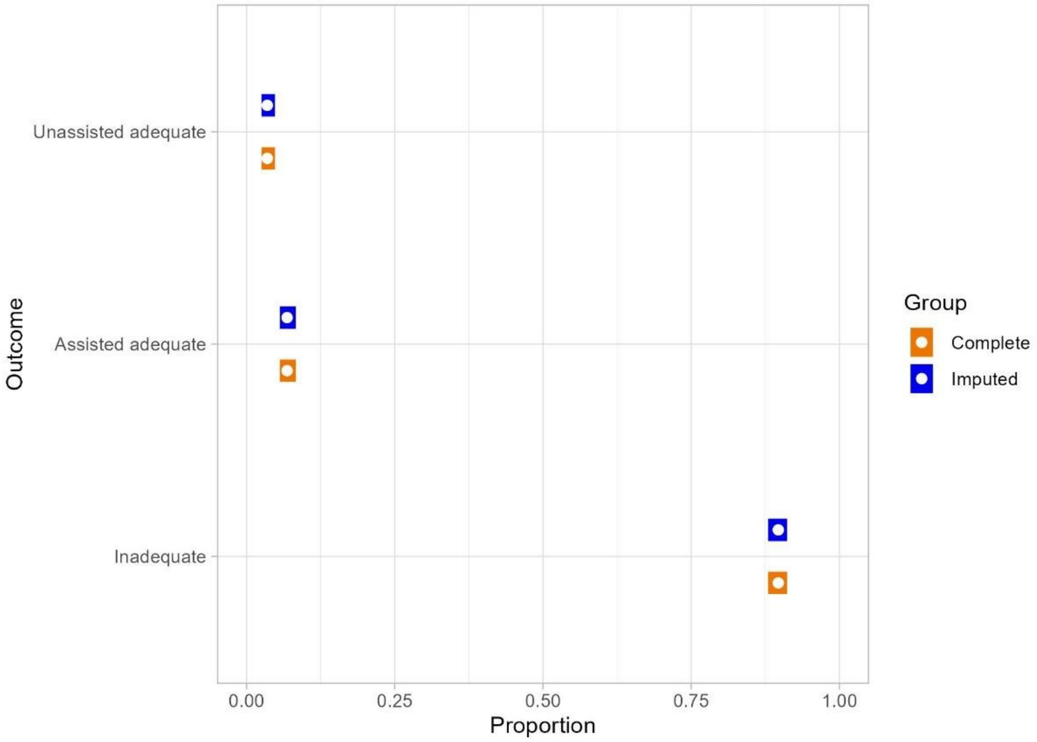

Supplementary Fig. 13: Plot showing the differences between point estimates and 95% confidence intervals in complete case and imputed data for urinary outcomes for patients who were not leak free and pad free at baseline.

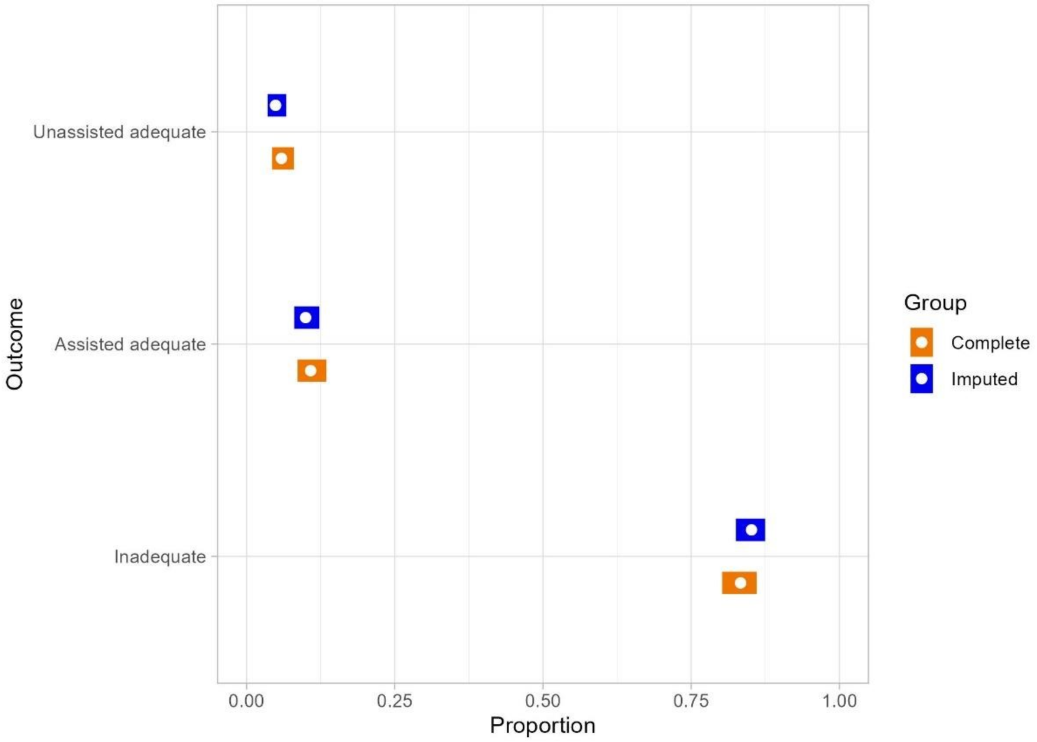

Supplementary Fig. 14 Plot showing the differences between point estimates and 95% confidence intervals in complete case and imputed data for urinary outcomes for patients who were not leak free and pad free at baseline.

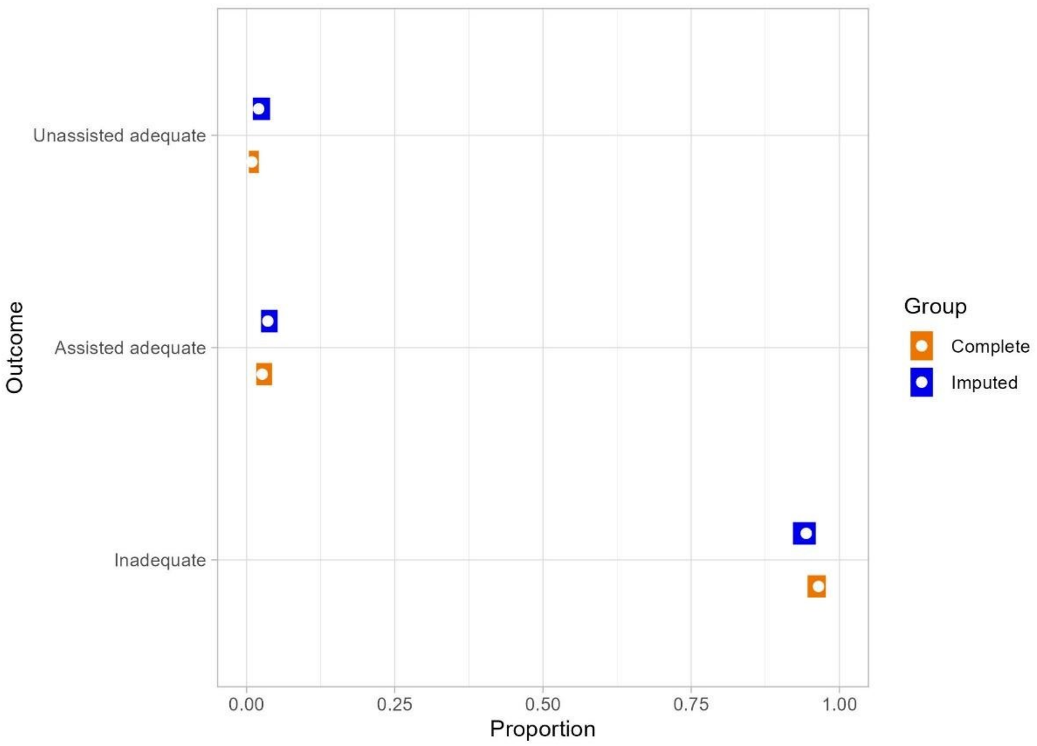

Supplementary Fig. 15 Plot showing the differences between point estimates and 95% confidence intervals in complete case and imputed data for urinary outcomes for patients who were not leak free and pad free at baseline.

Tables for missing data

Supplementary Table 1: Percentage of missing data for age, urinary incontinence and sexual function domain responses, and assistance use at each time point. These variables were used in the multiple imputation.

	**Baseline**	**Month 1**	**Month 3**	**Month 6**	**Month 12**	**Any**
Age	4%					
Over the past 4 weeks, how often have you leaked urine?	<1%	30%	30%	33%	30%	55%
Which of the following best describes your urinary control during the last 4 weeks?	<1%	30%	30%	32%	30%	55%
How many pads or adult diapers per day did you usually use to control leakage during the last 4 weeks?	<1%	30%	30%	32%	30%	54%
How big a problem, if any, has each of the following been for you during the last 4 weeks? Dripping or leaking urine	1%	30%	30%	32%	29%	54%
How would you rate each of the following during the last 4 weeks? Your ability to have an erection	3%	32%	34%	35%	33%	63%

How would you rate each of the following during the last 4 weeks? Your ability to reach orgasm (climax)	5%	33%	35%	36%	34%	65%
How would you describe the usual QUALITY of your erections during the last 4 weeks?	3%	31%	33%	34%	32%	60%
How would you describe the FREQUENCY of your erections during the last 4 weeks?	3%	32%	32%	34%	31%	58%
Overall, how would you rate your ability to function sexually during the last 4 weeks?	2%	31%	32%	33%	31%	57%
Overall, how big a problem has your sexual function or lack of sexual function been for you during the last 4 weeks?	2%	31%	31%	33%	31%	56%
Use of assistance for erections	2%	31%	32%	34%	32%	59%
Any	12%	36%	37%	38%	38%	71%

Supplementary Table 2: Percentage of missing data for the urinary incontinence and sexual function domain scores at each time point.

	**Baseline**	**Month 1**	**Month 3**	**Month 6**	**Month 12**	**Any**
**Urinary incontinence domain score**	**2%**	**30%**	**31%**	**33%**	**31%**	**57%**
**Sexual function domain score**	**4%**	**33%**	**34%**	**35%**	**33%**	**63%**
**Either**	**5%**	**33%**	**34%**	**36%**	**35%**	**65%**

Supplementary Table 3: Results of the multiple imputation. Percentages may not add to one hundred due to rounding.

**Urinary**	**All**	**Baseline leak and pad free**	**Not baseline leak and pad free**
Leak free	42% (95%CI: 39, 45)	47% (95%CI: 44, 50)	26% (95%CI: 22, 31)
Pad free	62% (95%CI: 60, 65)	66% (95%CI: 63, 69)	50% (95%CI: 45, 55)
Leak and pad free	36% (95%CI: 34, 39)	41% (95%CI: 39, 44)	20% (95%CI: 16, 24)
**Sexual**	**All**	**Baseline natural erections**	**Not baseline natural erections**
Unassisted adequate erections	3% (95%CI: 3, 5)	5% (95%CI: 4, 7)	2% (95%CI: 1, 4)
Assisted adequate erections	7% (95%CI: 6, 8)	10% (95%CI: 8, 12)	4% (95%CI: 2, 5)
Inadequate erections	90% (95%CI: 88, 91)	85% (95%CI: 83, 87)	94% (95%CI: 92, 96)

Statistical code

All statistical programming was performed using R 4.2.2.[3-21] Code is available on GitHub (https://github.com/JoshuaBridge/TrueNTHPostSurgery).

References

1. Gomes M, Gutacker N, Bojke C, Street A. Addressing Missing Data in Patient-Reported Outcome Measures (PROMS): Implications for the Use of PROMS for Comparing Provider Performance. *Health Economics* 2016; 25(5): 515-28.
2. Michigan Medicine. EPIC. 2015/05/12/T12:30:15-04:00 2015. https://medicine.umich.edu/dept/urology/research/epic.
3. Stef van Buuren, Karin Groothuis-Oudshoorn (2011). mice: Multivariate Imputation by Chained Equations in R. Journal of Statistical Software, 45(3), 1-67. DOI 10.18637/jss.v045.i03.
4. Heymans M (2022). miceafter: Data and Statistical Analyses after Multiple Imputation. R package version 0.5.0, https://CRAN.R-project.org/package=miceafter.
5. R Core Team (2023). R: A language and environment for statistical computing. R Foundation for Statistical Computing,nVienna, Austria. URL https://www.R-project.org/.
6. Henry L, Wickham H (2023). rlang: Functions for Base Types and Core R and 'Tidyverse' Features. R package version 1.1.1, https://CRAN.R-project.org/package=rlang.
7. Wickham H, François R, Henry L, Müller K, Vaughan D (2023). dplyr: A Grammar of Data Manipulation. R package version 1.1.3, https://CRAN.R-project.org/package=dplyr.
8. Xie Y (2023). knitr: A General-Purpose Package for Dynamic Report Generation in R. R package version 1.44, https://yihui.org/knitr/.

9. Yihui Xie (2015) Dynamic Documents with R and knitr. 2nd edition. Chapman and Hall/CRC. ISBN 978-1498716963

10. Yihui Xie (2014) knitr: A Comprehensive Tool for Reproducible Research in R. In Victoria Stodden, Friedrich Leisch and Roger D. Peng, editors, Implementing Reproducible Computational Research. Chapman and Hall/CRC. ISBN 978-1466561595

11. H. Wickham. ggplot2: Elegant Graphics for Data Analysis. Springer-Verlag New York, 2016.

12. Harrell Jr F (2023). Hmisc: Harrell Miscellaneous. R package version 5.1-0, https://CRAN.R-project.org/package=Hmisc.

13. Hofmann H, VanderPlas S, Ge Y (2022). ggpcp: Parallel Coordinate Plots in the 'ggplot2' Framework. R package version 0.2.0, https://CRAN.R-project.org/package=ggpcp.

14. James Honaker, Gary King, Matthew Blackwell (2011). Amelia II: A Program for Missing Data. Journal of Statistical Software, 45(7), 1-47. URL https://www.jstatsoft.org/v45/i07/.

15. Robitzsch, A., & Grund, S. (2023). miceadds: Some Additional Multiple Imputation Functions, Especially for 'mice'. R package version 3.16-18. https://CRAN.Rproject.org/package=miceadds

16. Gagolewski M (2022). “stringi: Fast and portable character string processing in R.” Journal of Statistical Software, *103*(2), 1-59. doi:10.18637/jss.v103.i02 https://doi.org/10.18637/jss.v103.i02.

17. Pedersen T (2023). patchwork: The Composer of Plots. R package version 1.1.3, https://CRAN.R-project.org/package=patchwork.

18. Gohel D, Skintzos P (2023). flextable: Functions for Tabular Reporting. R package version 0.9.3, https://CRAN.R-project.org/package=flextable.

19. Pedersen T, Robinson D (2022). gganimate: A Grammar of Animated Graphics. R package version 1.0.8, https://CRAN.R-project.org/package=gganimate.

20. Ooms J, Kornel Lesiński, Authors of the dependency Rust crates (2023). gifski: Highest Quality GIF Encoder. R package version 1.12.0-2, https://CRAN.Rproject.org/package=gifski.

21. Wickham H (2022). stringr: Simple, Consistent Wrappers for Common String Operations. R package version 1.5.0, https://CRAN.R-project.org/package=stringr.
